# Role of early bedside bronchoscopy-assisted sputum aspiration in the treatment of pneumonia in stroke patients

**DOI:** 10.3389/fmed.2026.1747464

**Published:** 2026-03-24

**Authors:** Xu He, Liyan Bo, Xinling Li, Chunhua Li, Hongzhu Bao, Dongbin Li, Liang Shi, Congcong Li

**Affiliations:** 1 Department of Respiratory and Critical Care Medicine, General Hospital of Northern Theater Command, Shenyang, Liaoning, China; 2 Department of Traditional Chinese Medicine, Shenyang Fourth People’s Hospital, Shenyang, Liaoning, China

**Keywords:** bronchoscopy, inflammation, pneumonia, sputum aspiration, stroke

## Abstract

**Objective:**

To evaluate the safety and efficacy of early bedside bronchoscopy-assisted sputum aspiration in stroke patients with pneumonia.

**Methods:**

Data from patients with stroke-associated pneumonia admitted to the Department of Respiratory and Critical Care Medicine, General Hospital of Northern Theater Command, were collected from January 1, 2022, to December 31, 2024. A retrospective analysis was conducted to assess the safety and efficacy of early bedside bronchoscopy-assisted sputum aspiration in these patients.

**Results:**

The length of ICU stay in the control group was significantly longer than that in the bronchoscopy treatment group (6.199 ± 11.38 vs. 3.364 ± 4.574; *p* = 0.0371), and the duration of antibiotic use in the control group was also significantly longer than that in the treatment group (12.51 ± 10.38 vs. 9.792 ± 7.17; *p* = 0.0414). The proportion of patients with clinical improvement was higher in the bronchoscopy-assisted sputum aspiration group than in the control group, but the difference between the two groups was not statistically significant. There was no significant difference in the incidence of complications such as hypoxia, hemoptysis, or arrhythmia between the two groups.

**Conclusion:**

The application of bedside fibreoptic bronchoscopy can reduce the length of ICU stay and the duration of antibiotic use in patients, and has important value in clinical practice.

## Highlights

This study provides a comprehensive assessment of the safety and efficacy of early bedside bronchoscopy-assisted sputum aspiration in stroke patients with pneumonia. The key findings are as follows:

- Bronchoscopy-assisted sputum aspiration can enhance the effect of anti-infective treatment and significantly shorten the length of ICU stay and duration of antibiotic use.
- Bronchoscopy-assisted sputum aspiration is safe for stroke patients.

## 1 Introduction

Stroke includes ischemic stroke and hemorrhagic stroke, with an increasing incidence annually, and it is among the major causes of disability (1). Stroke-induced immunosuppression syndrome (SIDS) and stroke-associated infections are important complications of stroke (2). Stroke-induced immunosuppression can not only reduce the inflammatory response and protect brain tissue by regulating the local and systemic immune systems, but also weaken the body’s resistance to pathogens, leading to infections (2). Infection is among the main causes of death in stroke patients and is closely related to the prognosis of patients and the occurrence of infectious complications (3). Among these complications, pneumonia is the most common type of infection after acute stroke (4).

Stroke often causes patients to stay in bed for a long time and impairs the cough reflex, resulting in the failure of convenient expectoration of respiratory secretions and the occurrence of hypostatic pneumonia (4). Stroke patients often experience disturbances in consciousness and abnormal swallowing reflexes, and the probability of aspiration is significantly greater in these patients than in healthy individuals, leading to aspiration pneumonia. Persistent pulmonary infection strongly affects the treatment and early rehabilitation of stroke patients, resulting in poor prognosis. Strengthening the drainage of airway secretions such as sputum and active anti-infective treatment are key measures for the treatment of stroke-associated pneumonia (5). In the ICU, bedside bronchoscopy is also often used for assisted sputum aspiration, alveolar lavage, and etiological specimen collection in such patients, but its importance has rarely been reported. In this study, the data of stroke patients with pneumonia who underwent bronchoscopy-assisted sputum aspiration in the intensive care ward of our department were statistically analyzed, and the safety and efficacy of bronchoscopy-assisted sputum aspiration in stroke patients were studied in detail.

## 2 Patients and methods

### 2.1 Research subjects

This study retrospectively analyzed the safety and efficacy of early bedside bronchoscopy-assisted sputum aspiration in stroke patients with pneumonia. We collected data from patients with stroke-associated pneumonia admitted to the Department of Respiratory and Critical Care Medicine, General Hospital of Northern Theater Command, from January 1, 2022, to December 31, 2024, including basic information such as sex, age, diagnosis, details of bronchoscopy-assisted sputum aspiration, occurrence of complications, and prognosis-related data such as length of hospital stay and duration of antibiotic use. This study was approved by the hospital ethics committee, with approval number Y (2024) No. 352.

The inclusion criteria were as follows: 1. A history of stroke or typical symptoms and signs; 2. A confirmed diagnosis of stroke by head CT or MRI; 3. Chest CT results suggesting the presence of pulmonary inflammation, meeting the indications for bronchoscopy to collect etiological specimens or for bronchoscopy-assisted sputum aspiration.

The exclusion criteria were as follows: 1. Other high-risk factors for pulmonary infection (such as malignant tumors, autoimmune diseases, immunodeficiency disorders, etc.); 2. Chest CT results indicating a high possibility of non-infectious inflammation; 3. Presence of contraindications to bronchoscopy-assisted sputum aspiration.

### 2.2 Data collation

Patients who met the inclusion criteria were divided into a control group and a bronchoscopy treatment group according to the sputum-aspiration methods received during hospitalization. The control group was given comprehensive treatment for pneumonia, including routine turning, mechanically assisted sputum excretion, sputum aspiration with a suction tube, and, when necessary, anti-infective drugs and expectorant drugs. Based on the treatment given to the control group, the treatment group received early bedside bronchoscopy-assisted sputum aspiration, and alveolar lavage was performed when necessary; the rest of the treatment was the same as that of the control group.

For antibiotic use, clinicians generally first selected antibiotics empirically according to the specific condition of patients (usually third-generation cephalosporins) and adjusted them to targeted anti-infective treatment as appropriate after the results of etiological tests, such as sputum smears and bacterial and fungal cultures, were reported.

In terms of sputum drainage methods, mechanically assisted sputum excretion and sputum aspiration with suction tubes were used in the control group when necessary. In accordance with the patients’ ability of spontaneous expectoration and the amount of sputum, patients were encouraged to expectorate sputum; when necessary, a sputum suction tube was used to aspirate the sputum in a timely manner. Sputum samples were collected for etiological testing on the first day of hospitalization.

Based on routine turning over, mechanical assisted sputum excretion, and sputum aspiration with a sputum suction tube, when necessary, the bronchoscopy treatment group received early (usually within 24 h) bedside bronchoscopy-assisted sputum aspiration, and sputum or bronchoalveolar lavage fluid was collected for etiological testing. All bedside bronchoscopy procedures were performed by attending physicians from the Department of Respiratory and Critical Care Medicine, each with at least 5 years of bronchoscopy experience. For bronchoscopy examination, patients received pharyngeal anesthesia with atomized lidocaine hydrochloride injection combined with dyclonine mucilage. The bronchoscope was inserted through the nose, tracheal intubation port, or tracheostomy and then gradually advanced into the lobar, segmental, and subsegmental bronchi to remove sputum and other substances until the lumen was unobstructed. If the sputum was too viscous to be aspirated, a small amount (usually approximately 5–10 mL) of normal saline was injected, followed by repeated negative-pressure suction until the sputum was completely removed. The frequency of bronchoscopy was usually once every two to 3 days.

Observation indicators: By reviewing medical records, we collected and compared the basic information, level of consciousness, respiratory support method, outcome, duration of antibiotic use, length of hospital stay, length of ICU treatment, and occurrence of adverse reactions during or after treatment between the two groups.

### 2.3 Statistical methods

**Figure 1:**
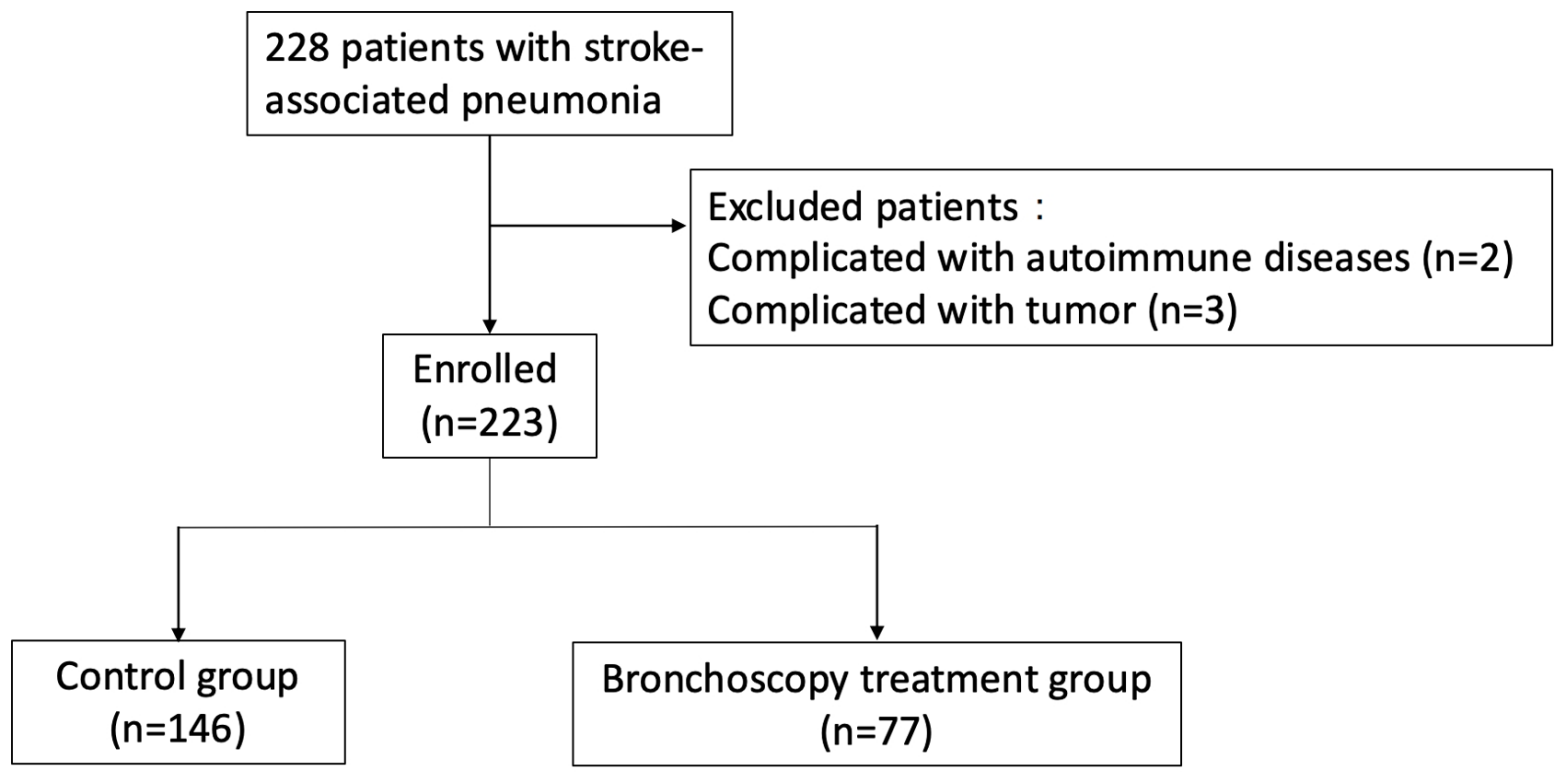
The flow chart of enrollment.

First, G*Power software was used for power analysis. Under the preset conditions of medium effect size (*d* = 0.5) and 0.8 test power, the estimated sample size was 128 cases. The data of the enrolled patients are expressed as the mean ± standard deviation (x̄±s). GraphPad Prism 8 software was used for data analysis. One-way ANOVA was used to analyze the differences between groups for continuous measurement data (such as age and length of hospital stay), and the χ^2^ test was used to analyze the differences between groups for count data (such as sex and complications). A *p* < 0.05 indicated that the difference was statistically significant (Figure 1).

## 3 Results

### 3.1 Basic patient information

A total of 223 patients with stroke-associated pneumonia admitted to our department from January 1, 2022, to December 31, 2024, were included. The average age was 72.48 ± 10.05 years, and the sample included 174 males and 49 females. There were 21 patients with cerebral hemorrhage, accounting for 9.42%, and 202 patients with cerebral infarction, accounting for 90.58%. The control group included 146 patients, and the bronchoscopy treatment group included 77 patients. The specific information is shown in Table 1.

**Table 1:** Demographic information of patients.

Item	Control group	Bronchoscopy treatment group	Statistical analysis	*p*-value
Male	113	61	χ^2^ = 0.09777	0.7545
Female	33	16		
Age (years)	73.05 ± 9.44	71.38 ± 11.11	*t* = 1.186, df = 221	0.2368
Consciousness status				
Conscious	132	66	χ^2^ = 1.117	0.2905
Unconscious	14	11		
Stroke type				
Cerebral hemorrhage	12	9	χ^2^ = 0.7112	0.3990
Cerebral infarction	134	68		
Respiratory support method				
Oxygen inhalation	115	51	χ^2^ = 11.63	0.003
Non-invasive ventilator	19	23		
Invasive ventilator	24	24		

### 3.2 Effects of bronchoscopy-assisted sputum aspiration on the length of hospital stay and duration of antibiotic use in stroke patients with pneumonia

The total length of hospital stay, length of ICU stay, and duration of antibiotic use were statistically analyzed between the two groups. The results (as shown in Table 2) revealed that the length of ICU stay in the control group was significantly longer than that in the bronchoscopy treatment group (6.199 ± 11.38 vs. 3.364 ± 4.574, *p* = 0.0371), and the duration of antibiotic use in the control group was also significantly longer than that in the treatment group (12.51 ± 10.38 vs. 9.79 ± 7.17, *p* = 0.0414). These results indicate that bronchoscopy-assisted sputum aspiration can reduce local inflammation, enhance the effect of anti-infective treatment, and significantly shorten the length of ICU stay and duration of antibiotic use, and its therapeutic effect is better than that of vibration sputum excretion and conventional sputum suction tubes.

**Table 2:** Effects of bronchoscopy-assisted sputum aspiration on the length of hospital stay and duration of antibiotic use.

Item	Control group	Bronchoscopy treatment group	Statistical analysis	*P*-value
Length of hospital stay	13.30 ± 11.35	11.17 ± 10.18	*t* = 1.381, df = 221	0.1687
Length of ICU stay	6.199 ± 11.38	3.364 ± 4.57	*t* = 2.097, df = 221	0.0371
Duration of antibiotic use	12.51 ± 10.38	9.79 ± 7.17	*t* = 2.051, df = 221	0.0414

### 3.3 Effect of bronchoscopy-assisted sputum aspiration on the outcomes of stroke patients with pneumonia

We also analyzed the effect of bronchoscopy-assisted sputum aspiration on the outcomes of stroke patients with pneumonia. As shown in Table 3, compared with the control group, the proportion of patients who improved was greater in the bronchoscopy-assisted sputum aspiration group, but the difference between the two groups was not statistically significant, which may be related to the more severe condition of patients in the bronchoscopy treatment group (the proportion of mechanical ventilation was significantly higher than that in the control group). Further logistic regression analysis revealed that the improvement trend in the bronchoscopy treatment group did not reach statistical significance (OR = 0.794, *p* = 0.627), and the use of ventilators did not significantly reduce the probability of improvement (OR = 1.391, *p* = 0.496).

**Table 3:** Effects of bronchoscopy-assisted sputum aspiration on the outcomes of stroke patients with pneumonia.

Patient outcome	Control group	Bronchoscopy treatment group	Statistical analysis	*P*-value
Improved	128	69	χ^2^ = 0.6288, df = 2	0.7302
Deteriorated	11	6		
Died	7	2		

### 3.4 Occurrence of complications of bronchoscopy-assisted sputum aspiration

Complications that may be caused by bronchoscopy-assisted sputum aspiration include transient hypoxia, hemoptysis, and arrhythmia. Among the patients included in this study, 19 had a transient decrease in oxygen saturation, including 13 in the control group and 6 in the bronchoscopy treatment group, with no significant difference between the two groups; 19 had blood-tinged sputum, including 12 in the control group and 7 in the bronchoscopy-assisted sputum aspiration group, and statistical analysis revealed no significant difference between the two groups. No malignant arrhythmia or other complications occurred (Table 4).

**Table 4:** Occurrence of complications of bronchoscopy-assisted sputum aspiration.

Item	Control group	Bronchoscopy treatment group	Statistical analysis	*P*-value
Hypoxia	13	6	χ^2^ = 0.07996	0.7773
Hemorrhage	12	7	χ^2^ = 0.04915	0.8245
Malignant arrhythmia	0	0	NA	NA
Others	0	0	NA	NA

### 3.5 Subgroup analyses based on stroke types

To further explore the effects of bronchoscopy-assisted sputum aspiration on different stroke types of patients, we conducted subgroup analyses. As shown in Table 5, for cerebral infarction patients, the length of ICU stay in the control group was significantly longer than that in the bronchoscopy treatment group (6.50 ± 11.77 vs. 3.38 ± 4.63, *p* = 0.04). However, for cerebral hemorrhage patients, we did not find similar differences. And this might be due to the relatively fewer patients with cerebral hemorrhage included in this study.

**Table 5:** Subgroup analyses based on stroke types.

Item	Cerebral hemorrhage (*N* = 21)			Cerebral infarction (*N* = 202)		
	Control group (*N* = 12)	Bronchoscopy treatment group (*N* = 9)	*P*-value	Control group (*N* = 134)	Bronchoscopy treatment group (*N* = 68)	*P*-value
Length of hospital stay	12.67 ± 18.50	9.22 ± 9.82	0.40	13.36 ± 11.60	11.43 ± 10.27	0.25
Length of ICU stay	2.83 ± 4.24	3.22 ± 4.38	0.84	6.50 ± 11.77	3.38 ± 4.63	0.04

## 4 Discussion

In this study, we statistically analyzed the data of stroke patients who received early bedside bronchoscopy-assisted sputum aspiration in the intensive care ward of our department. By comparing and analyzing the efficacy and occurrence of complications between conventional sputum suction catheters and bronchoscopy-assisted sputum aspiration, we clarified the safety and efficacy of bronchoscopy-assisted sputum aspiration in stroke patients.

Stroke patients may experience acute changes in intracranial pressure and severe cerebral edema, which make them prone to cerebral ischemia and hypoxia and decreased immunity (2, 3). In addition, patients are in a comatose state with severe disturbances of consciousness and are prone to complications such as pulmonary infection (6, 7). Bronchoscopy can smoothly pass through the nasal cavity, oral cavity, and other parts and enter the trachea and main bronchi at all levels on both sides under direct vision, which is widely used in the diagnosis and treatment of respiratory diseases. With the assistance of bronchoscopy, sputum and secretions in the patient’s airway can be removed more effectively and accurately (8). Previous studies on severe pneumonia have also shown that fibreoptic bronchoscopy and bronchoalveolar lavage are helpful for identifying the causes of pulmonary infection, effectively dredging the bronchi, and can be used as tools to evaluate lung function (9, 10). Moreover, fibreoptic bronchoscopy lavage can also alleviate the respiratory symptoms of patients. Bronchoscopic sputum aspiration allows directly observation of the sputum location and, compared with general sputum aspiration, avoids mucosal damage caused by the blind use of negative pressure (11). In addition, bronchoalveolar lavage (BAL) can be used to dilute sputum, making it easier to aspirate, and repeated lavage with normal saline can reduce inflammatory factors, alleviate local inflammation, and enhance the effect of anti-infective treatment (12, 13). Studies on severe pneumonia have shown that on the basis of conventional treatment, fibreoptic bronchoscopic sputum aspiration and lavage can significantly shorten ICU stay and the duration of mechanical ventilation, and the therapeutic effect is better than that of vibration sputum excretion and conventional sputum suction tubes (14, 15).

Stroke-associated pneumonia (SAP) refers to pneumonia occurring in patients with acute stroke. Its symptoms are similar to those of ordinary pneumonia, including fever, cough, expectoration, and dyspnea (16). As one of the most common complications of stroke, SAP is characterized by high incidence and mortality rates. In mild cases, it leads to prolonged hospital stays and increased medical costs; in severe cases, it can even cause patient death (5, 17). Persistent pulmonary infection strongly affects the treatment and early rehabilitation of stroke patients, resulting in poor prognosis (12). The pathogenesis of SAP is associated with factors such as prolonged bed rest, a weakened cough reflex, aspiration, bacterial colonization, impaired respiratory defense capabilities, and reduced body immunity (2, 3). A weakened cough reflex prevents the excretion of respiratory secretions, thereby causing hypostatic pneumonia. In addition, disturbances in consciousness and abnormal swallowing reflexes in stroke patients often lead to aspiration, which in turn triggers aspiration pneumonia. Moreover, prolonged treatment in the intensive care unit (ICU) for stroke patients is often accompanied by nosocomial infections, leading to pulmonary inflammation caused by drug-resistant bacterial infections (4, 18).

Strengthening the drainage of airway secretions (such as sputum) and providing active anti-infective treatment are key measures for the management of SAP. In clinical practice, conventional expectorant drugs and antibiotics often fail to effectively control infections. In some cases, repeated infections and the use of antibiotics may even lead to the emergence of drug-resistant bacteria. Traditional sputum suction catheters cannot reach the distal bronchi and are unable to distinguish between lesioned and normal areas, which may cause further damage to the respiratory tract. In contrast, fibreoptic bronchoscopes can access the lumens of 3rd- to 4th-order bronchi and reach the bronchi with lesions in a targeted manner (19). For atelectasis caused by bronchial obstruction due to sputum or blood clots, fibreoptic bronchoscopes can be used to relieve airway obstruction quickly through irrigation and suction (8). This helps improve pulmonary ventilation and gas exchange functions and correct hypoxic states (8). Furthermore, bronchoalveolar lavage can dilute airway secretions, stimulate coughing to facilitate their excretion, and simultaneously clear the airway while eliminating allergens, inflammatory cells, and inflammatory mediators (20).

In this study, we focused on observing the clinical efficacy of bedside bronchoscopy-assisted sputum aspiration and bronchoalveolar lavage as adjuvant treatments for stroke patients with pneumonia. Our findings indicated that bronchoscopy-assisted sputum aspiration can reduce local inflammation, increase the efficacy of anti-infective treatment, and significantly shorten ICU stay and duration of antibiotic use. Its therapeutic effect was superior to that of vibration-assisted sputum excretion and conventional sputum suction catheters. Moreover, no serious adverse reactions occurred. In the comparison of prognosis between the two groups, the proportion of patients who improved in the bronchoscopy-assisted sputum aspiration group was higher than that in the control group; however, the difference between the two groups was not statistically significant. This phenomenon may be attributed to two main factors. First, the patients in the bronchoscopy treatment group had more severe conditions, as evidenced by the significantly higher proportion of ventilator use in this group than in the control group, indicating a more critical state of illness. Second, the sample size of this study was relatively small. Further studies may provide deeper insights into this issue.

There are some limitations needed to be clarified. First, as this is a retrospective study, group allocation might be subject to selection bias, particularly since patients in the bronchoscopy group tended to be more severely ill (e.g., higher proportion requiring mechanical ventilation). Second, the bronchoscopy group only included 77 patients, which might be not sufficient to determine the incidence of complications. Third, a well-designed RCT include stricter patient enrollment criteria, standardized intervention protocols and some more outcomes (such as inflammatory biomarker levels, long-term follow-up data) might provide a more comprehensive evaluation of the intervention.

## 5 Conclusion

In conclusion, by comparison with the use of conventional sputum suction catheters, we investigated the efficacy of bedside bronchoscopy-assisted sputum aspiration and bronchoalveolar lavage in the treatment of stroke patients with pneumonia. The application of bedside fibreoptic bronchoscopy resulted in improvements in both ICU stay and the duration of antibiotic use in patients, demonstrating its important value in clinical practice.

## Data Availability

The raw data supporting the conclusions of this article will be made available by the authors, without undue reservation.
